# Revitalising the medical emergency team call

**DOI:** 10.1186/cc14489

**Published:** 2015-03-16

**Authors:** KP Verma, S Jasiowski, K Jones

**Affiliations:** 1 Melbourne Health, Melbourne, Australia; 2 Ballarat Health Services, Ballarat, Australia

## Introduction

Medical emergency team (MET) calls are quickly becoming an integral part of the response to a deteriorating patient in Australia. Conceptually the MET call response incorporates a structured approach, but in practice this can quickly disintegrate. This collapse of method can leave patients without clear treatment plans and staff disenfranchised. We sought to improve the process of the MET call response at our regional hospital by introducing targeted interventions focused on teamwork, communication, leadership and role allocation.

## Methods

We invited junior doctors and nurses to complete a survey designed by a multidisciplinary MET Call Working Group; 138 staff (40% of population) completed the survey. Based on analysis of responses, a focused three-pronged intervention was formulated and implemented hospital wide. The arms of the intervention were: identification of the name and role of each staff member using highly visible labels; role allocation according to policy written through a multidisciplinary working group; and a time out during the response allowing a structured synopsis of the patient's current status to be communicated to the team. The intervention was preceded by extensive staff education, and 175 staff (50%) completed the survey 6 months later to assess its success.

## Results

The intervention significantly increased satisfaction amongst staff regarding: identification of the team leader and other key staff members at the response; and time out effectiveness in reducing repetition and improving staff understanding of the patient's status and medical issues. We found no significant change in staff perceptions regarding the clarity of the ongoing treatment plan at the end of the MET call response.

## Conclusion

Utilising a low-cost intervention in a regional setting, we were successful in improving staff perceptions of role allocation and communication within our MET call responses. The intervention also led to significantly increased overall satisfaction with the MET call system. Through our surveys we have identified other facets of the MET call response that also require attention. Given our encouraging

## Results

we are designing a follow-up intervention incorporating structured multidisciplinary training in MET call scenarios.

